# Giant *De Novo* Pleomorphic Adenoma Arising from the Parapharyngeal Space

**DOI:** 10.1155/2013/742910

**Published:** 2013-12-12

**Authors:** Sang Hwang, Sim Choroomi, Ben McArdle, Ian Jacobson

**Affiliations:** ^1^Department of Otolaryngology and Head and Neck Surgery, Prince of Wales Hospital, Randwick, NSW 2031, Australia; ^2^Prince of Wales Hospital Clinical School, University of New South Wales, Kensington, NSW 2052, Australia

## Abstract

*Introduction*. *De novo* pleomorphic adenomas in the parapharyngeal space are rare and cause difficulties in its surgical management. We report the largest *de novo* pleomorphic adenoma arising from the parapharyngeal space and discuss its surgical management. *Presentation of Case*. A 34-year-old male presented with a giant *de novo* pleomorphic adenoma arising from the parapharyngeal space, which was initially misdiagnosed as an impacted wisdom tooth. Measuring 8.4 × 6.5 × 3.9 cm in size and weighing 87.3 g, this is the largest primary salivary gland tumour arising *de novo* from the parapharyngeal space reported in the literature, presenting challenges in its surgical management. *Discussion*. Parapharyngeal space tumours cause nonspecific symptoms and may be difficult to diagnose, which can allow the tumours to become very large and cause obstructive and compressive symptoms in an anatomically difficult area. A combined trans-cervical and trans-oral approach can be used to safely perform an *en bloc* resection. *Conclusion*. We report the diagnosis and surgical management of the largest pleomorphic adenoma arising *de novo* from the parapharyngeal space reported in the literature.

## 1. Introduction

The parapharyngeal space is found anterior to the cervical column, posterior to the infratemporal fossa, and laterally to the nasopharynx. It forms an inverted pyramid with the skull base superiorly and the apex at the joint between the posterior belly of the digastric muscle and the greater cornu of the hyoid bone [1].

Tumours arising *de novo *in the parapharyngeal space are very rare and present challenges in achieving *en bloc* excision without spilling the contents of the tumour [1]. We describe the largest primary salivary gland tumour arising *de novo* from the parapharyngeal space and discuss its management.

## 2. Case History

A 34-year-old male presented to our multidisciplinary head and neck clinic with a 3-year history of recurrent discomfort in his right retromolar trigone. These episodes were initially diagnosed by a dentist as an impacted wisdom tooth with subclinical infection and were treated with oral antibiotics with minor improvement in symptoms. One year prior to presentation, the patient noticed a prominence on the right side of his oropharynx but this was not reviewed by a medical professional.

Over the last 2 years, the patient had worsening obstructive symptoms, including snoring, episodes of sleep apnoea, nasal speech, and a sensation of decreased hearing in his right ear. The patient also reported 7 kg of weight loss and lethargy in the preceding 3 months before presentation. There was no relevant past medical or family history and the patient took no regular medications. He was a lifelong nonsmoker with occasional consumption of alcohol.

**Figure 1:**
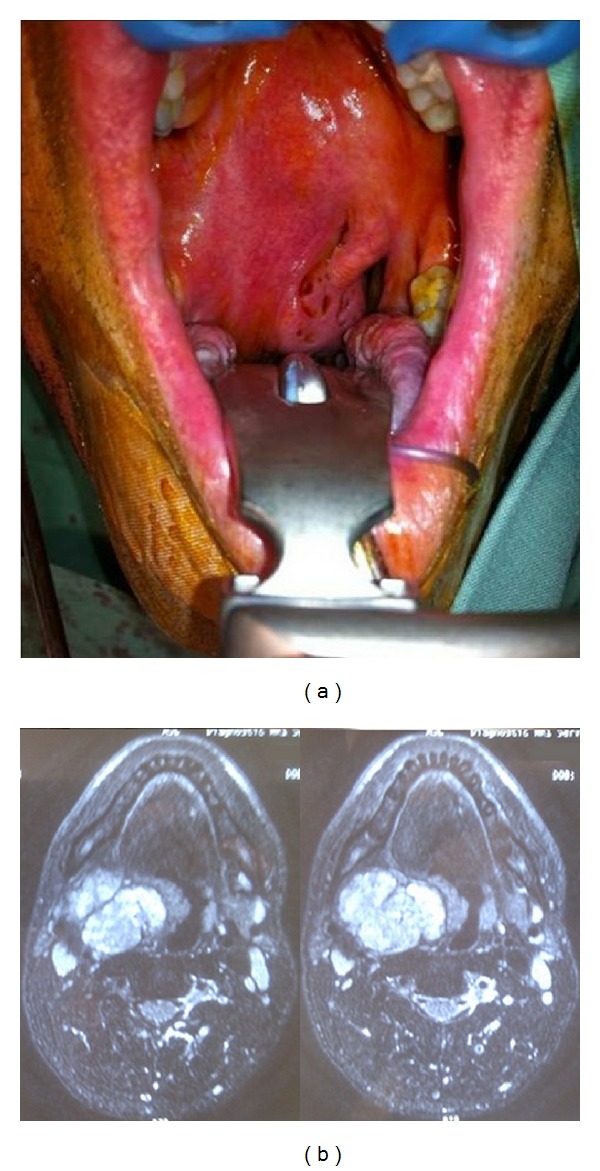
(a) The soft palate is displaced anteromedially by the parapharyngeal mass. (b) Magnetic resonance imaging of the parapharyngeal mass showing a lobulated, homogeneous mass.

Physical examination revealed a submucosal mass arising from the lateral wall of the right oropharynx, displacing the soft palate and the right anterior tonsillar pillar anteromedially (Figure 1(a)). The mass crossed the midline, narrowing the right oropharyngeal inlet and filling the nasopharynx, causing serous otitis media. Nasoendoscopy defined a mass that extends along the right lateral pharyngeal wall superiorly to the base of skull and inferiorly to the vallecula but not involving the right glossotonsillar sulcus or the posterior 1/3rd of the tongue. There were no lymphadenopathy or cranial nerve deficits.

Magnetic resonance imaging (MRI) demonstrated a well demarcated but lobulated homogenous mass with little contrast enhancement, separate from the deep lobe of the parotid (Figure 1(b)). There were no features to suggest tumour necrosis. A fine needle aspiration biopsy showed features consistent with a salivary gland tumour.

**Figure 2:**
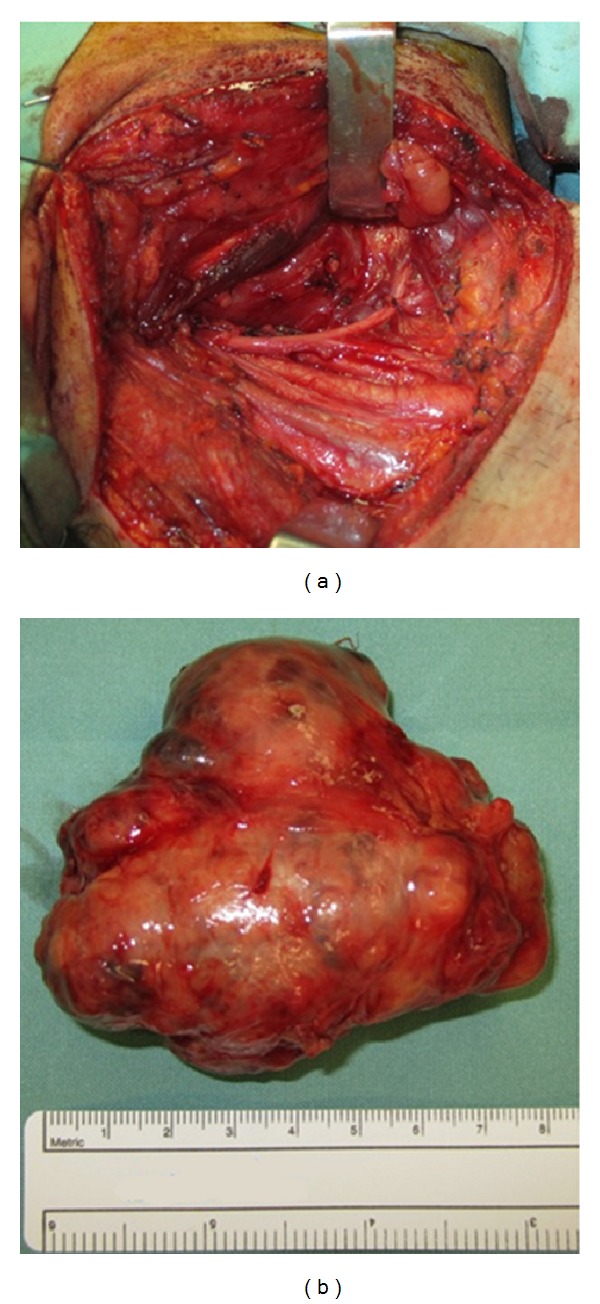
(a) The transcervical exposure of the tumour is demonstrated. (b) The excised specimen.

Surgical excision was performed via a combined transoral and transcervical approach, avoiding the need to split his mandible. A linear incision over the patient's right neck and a linear paramedian incision over the soft palate were used and an extracapsular blunt dissection was performed, and the tumour was delivered via the cervical neck *en bloc* (Figure 2(a)).

One week after-operation, the patient developed a deep space collection of the neck which was surgically drained and treated with intravenous antibiotics with good effect. The patient is well at 3 months from the initial operation.

Pathology revealed a pleomorphic adenoma of the salivary gland, 8.4 × 6.5 × 3.9 cm in size and weighing 87.3 g (Figure 2(b)). A literature search reveals this to be the largest primary salivary gland tumour arising *de novo* from the parapharyngeal space.

## 3. Discussion

Parapharyngeal space tumours are rare accounting for only 0.5% of head and neck neoplasms. Up to 80% are benign and 40–50% originate from the salivary glands, with pleomorphic adenoma being the most common [2]. They are often large at the time of presentation as they may be asymptomatic or misdiagnosed when being small [1].

Large parapharyngeal space tumours have been previously reported in literature, but these tumours often originate from the deep lobe of the parotid gland, which extends into the parapharyngeal space through the stylomandibular tunnel [2, 3]. It is rare to diagnose *de novo* pleomorphic adenoma in the parapharyngeal space [4], and it has been previously suggested that this may be secondary to displaced or aberrant salivary gland tissue [5].

There are numerous external (including transcervical and transparotid) methods to surgically access and perform an extracapsular dissection of parapharyngeal space tumours [1, 4]. In large tumours, this may be combined with a transoral approach, which has been described for excising small, nonvascular neoplasms that originate in the prestyloid compartment of the parapharyngeal space [1].

In this case, a combined trans-cervical and trans-oral approach was used to dissect out the tumour, which was delivered through the cervical incision. This is an effective way of excising giant parapharyngeal space tumours *en bloc*, maintaining oncological planes and minimizing the risk of spillage of the contents of the tumour.

In summary, we report a giant pleomorphic adenoma arising *de novo* from the parapharyngeal space. The combined trans-cervical and trans-oral surgical approach was successfully used in this anatomically difficult area and may be considered for excision of such giant tumours in the parapharyngeal space.
